# Laparoscopic exploration of a wandering spleen in a complex adolescent case with sigmoid volvulus and left-side portal hypertension: a case report

**DOI:** 10.1093/jscr/rjae059

**Published:** 2024-02-13

**Authors:** Ahmed Rafei, Nadir Ali Hilal

**Affiliations:** National Center for Gastrointestinal and Liver Diseases, Khartoum 15004, Sudan; National Center for Gastrointestinal and Liver Diseases, Khartoum 15004, Sudan

**Keywords:** wandering spleen, laparoscopic splenectomy, sigmoid volvulus, portal hypertension, Sudan

## Abstract

Wandering spleen (WS) is a rare condition characterized by the hypermobility of the spleen due to the absence or abnormal flexibility of suspensory ligaments. We present a 16-year-old female presented with intermittent abdominal pain, constipation, and a palpable mass in the right iliac fossa. Imaging revealed a WS associated with sigmoid volvulus and portal hypertension. Despite a decade of symptoms, the patient remained undiagnosed. Laparoscopic splenectomy was performed successfully, addressing both WS and sigmoid volvulus. The patient’s symptoms resolved, and she was discharged in good condition. This case emphasizes the need for clinical awareness of WS in the differential diagnosis of abdominal pain. It highlights the role of imaging in prompt diagnosis and the necessity of surgical intervention. Our case sheds light on the association of WS with other conditions, providing clinicians with valuable insights for effective management.

## Introduction

The spleen typically resides in the left hypochondriac region of the abdomen, where it is held in position by various suspensory ligaments, including the gastrosplenic ligament, the splenorenal ligament, and the phrenicocolic ligament [1]. In rare occasions, it can be found in other locations, a phenomenon referred to as a wandering spleen (WS). Splenic hypermobility, the hallmark of WS, is a rare clinical condition caused by the absence or abnormal flexibility of these suspensory ligaments, which are responsible for holding the spleen in place, allowing it to migrate to any part of the abdomen or pelvis [2].

Torsion is the main complication associated with a WS, often presenting as acute, chronic, or intermittent abdominal symptoms. Alternatively, WS may present as an asymptomatic, palpable abdominal mass [3]. Here, we present a case of a young female with intermittent abdominal pain and a right iliac mass confirmed as WS associated with sigmoid volvulus and portal hypertension.

## Case presentation

A 16-year-old Sudanese female presented with a 3-month history of intermittent, vague abdominal pain. She recounted experiencing sudden-onset, intermittent abdominal pain since the age of 6. Additionally, she reported symptoms of constipation, abdominal discomfort, along with fatigue and weight loss.

Despite multiple doctor visits, the patient had no history of hospitalization or blood transfusion. She was on long-term medication for supplements, and analgesia, with no history of similar conditions, genetic disorders, or congenital anomalies.

Clinical examination revealed a slightly distended abdomen. A mobile tender mass, was palpated in the right iliac fossa, soft in consistency. The left upper quadrant was empty, and the liver size was within normal limits. No other palpable masses were noted. Laboratory findings were normal.

**Figure 1:**
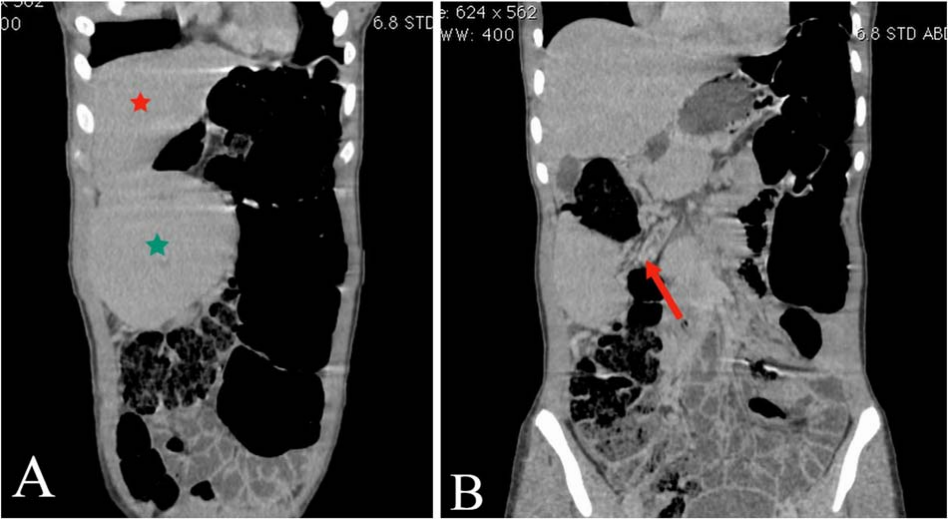
(A and B) The coronal plane of the abdominal CT reveals the liver (A, red star) and the WS in the right upper quadrant (A, green star), with torsion evident in the splenic mesentery (B, arrow).

**Figure 2:**
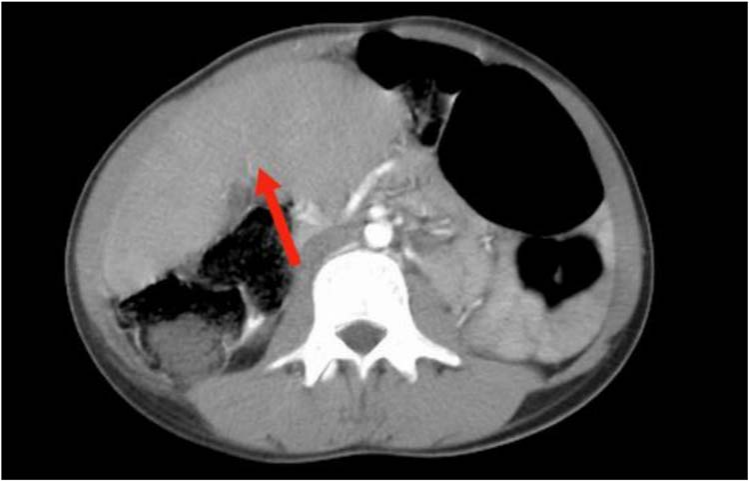
The axial plane of the abdominal CT reveals the spleen in the right upper quadrant.

Abdominal ultrasound revealed the spleen was seen in the right lumbar region, displaying mild splenomegaly. The spleen exhibited normal texture and good vascularity. A CT scan, revealed the absence of the spleen in the left upper quadrant and confirmed the diagnosis of a WS (Figs 1 and 2). We also identified elements of splenic pedicle torsion (Fig. 1B), an associated sigmoid volvulus, and dilated tortuous short gastric vessels, indicative of left-side portal hypertension.

**Figure 3:**
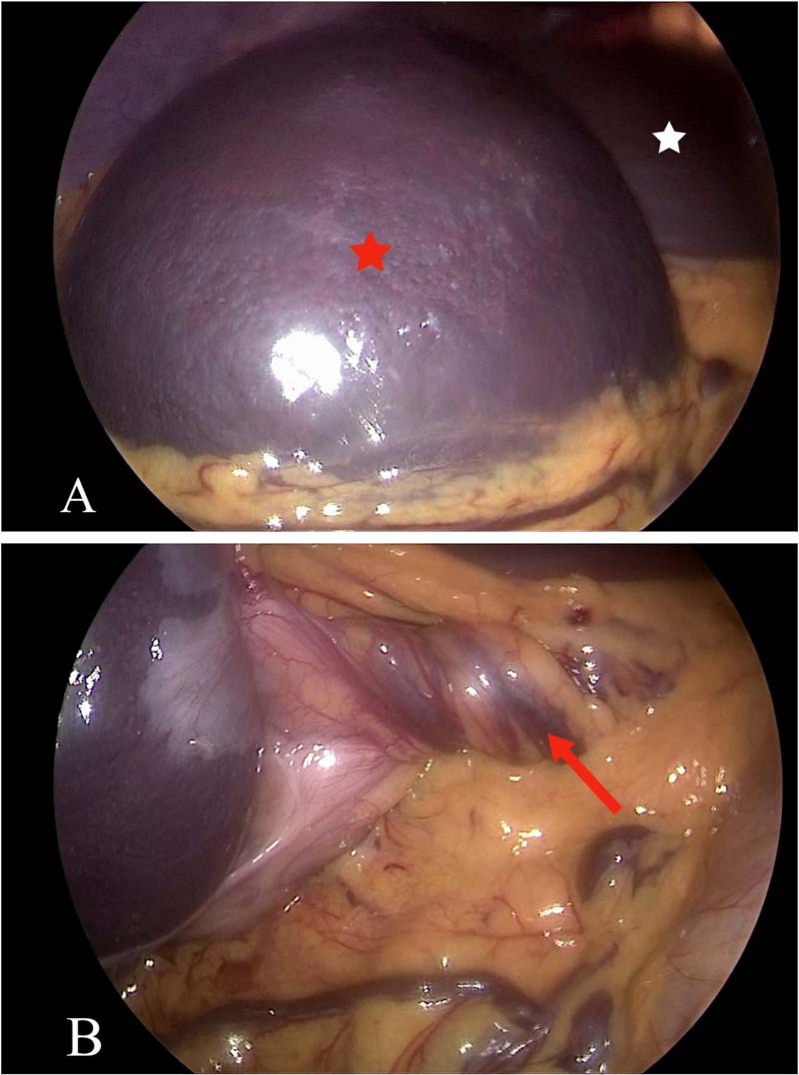
(A and B) The laparoscopic view shows the spleen (A, red star) and the liver (A, white star), revealing torsion in the spleen mesentery (B, arrow).

Laparoscopic splenectomy was done, involved the use of a 10-mm umbilical port and a 30° camera. The spleen was visualized on the right side below the liver (Fig. 3A), with complete torsion of the pedicle (Fig. 3B). Two working ports were established in the left iliac fossa and left hypochondria. The pedicle was detorted to ensure that the pancreas tail was away. The splenic artery and vein were ligated separately using Hemolock, and hemostasis was secured. The spleen was subsequently removed through a Pfannenstiel incision. Additionally, the sigmoid colon was identified in the left upper quadrant, with bands causing partial volvulus. These bands were released, and the distension was relieved. The patient was discharged in good condition. Two weeks later, she received her vaccinations and then followed up for 8 months postoperatively.

## Discussion

WS, also known as splenoptosis, ectopic spleen, or floating spleen, is a rare condition caused by the absence or abnormal flexibility of spleen’s suspensory ligaments [1]. The prevalence of this condition is estimated to be 0.2%, with fewer than 500 reported cases in the existing literature [4].

Furthermore, the etiology of this disorder may arise from congenital or acquired factors. Although the pathophysiological process behind congenital WS remains unclear, many medical studies connect it to the improper development of the mesogastrium dorsum during embryonic life [5]. The acquired variant usually manifests in women of childbearing age, influenced by maternal hormones. Research indicates that both estrogen and progesterone contribute to the elevated activity of relaxin receptors on cells, thereby promoting increased ligamentous laxity [6].

The splenic vascular pedicle, comprising the splenic artery and six additional branches of the splenic vein, may undergo elongation and subsequent twisting, resulting in congestion and splenomegaly. This pathological enlargement can further exacerbate the hypermobility of the spleen. The intermittent abdominal pain faced by our patient for the past decade may result from intermittent torsion and spontaneous detorsion of the splenic pedicle. Interestingly, our patient was misdiagnosed for 10 years. Depending on the degree of torsion, this condition may lead to various complications, including splenic infarction, gangrene, abscess formation with peritonitis, intestinal obstruction, and spleen rupture [7]. Moreover, if the tail of the pancreas is partially twisted alongside the vascular pedicle at the splenic hilum, it has the potential to induce pancreatitis and ischemia of the pancreatic tail. Additionally, the mass effects of the WS on adjacent structures can lead to complications, including intestinal obstruction, gastric volvulus, and pancreatitis [2]. There are a few reported cases describing the association of a WS with portal hypertension and fundal varices; however, cases of mesenteric varices are extremely rare [8].

Given the nonspecific nature of clinical presentation and laboratory studies, the diagnosis primarily relies on imaging examinations. Both ultrasonography and computed tomography play crucial roles, as they can provide valuable insights for the diagnosis and preoperative assessment of WS, along with any associated conditions.

Surgical intervention stands as the sole recommended approach for managing a WS. Splenectomy is often necessary in cases involving splenic torsion with infarction, and it may be recommended for significantly enlarged WSs that are not suitable for splenopexy [9]. In certain scenarios, preserving the spleen through splenopexy is preferred to reduce the potential risks of overwhelming postsplenectomy sepsis [10]. Even in asymptomatic cases, surgical treatment is considered to be necessary due to the substantial rate of complications [9]. Conservative management is not advisable, as it is linked to a complication rate exceeding 50% [4].

## Conclusion

WS, a rare medical condition, presents a diagnostic challenge requiring a heightened level of clinical suspicion. This case highlights the importance of considering WS in the differential diagnosis of abdominal pain, especially in patients with a long history of intermittent abdominal symptoms. Imaging studies facilitates a prompt confirmation of the diagnosis and identification of potential complications. Surgical intervention remains the definitive treatment for WS due to the high risk of complications associated with conservative management.

## References

[1] Jawad M , Yusuf MH, Al Doaibel KA, et al. Wandering spleen: a rare case from the emergency department. Cureus 2023;15:e33246. 10.7759/cureus.33246. 36741617 PMC9890613

[2] Flores-Ríos E , Méndez-Díaz C, Rodríguez-García E, et al. Wandering spleen, gastric and pancreatic volvulus and right-sided descending and sigmoid colon. J Radiol Case Rep 2015;9:18–25. 10.3941/jrcr.v9i10.2475. PMC4638397 26629290

[3] Safioleas MC , Stamatakos MC, Diab AI, et al. Wandering spleen with torsion of the pedicle. Saudi Med J 2007;28:135–6. 17206307

[4] Masroor M , Sarwari MA. Torsion of the wandering spleen as an abdominal emergency: a case report. BMC Surg 2021;21:289. 10.1186/s12893-021-01289-x. 34107944 PMC8190838

[5] Allen KB , Gay BB Jr, Skandalakis JE. Wandering spleen: anatomic and radiologic considerations. South Med J 1992;85:976–84. 10.1097/00007611-199210000-00011. 1411739

[6] Dehghan F , Haerian BS, Muniandy S, et al. The effect of relaxin on the musculoskeletal system. Scand J Med Sci Sports 2014;24:e220–9. 10.1111/sms.12149. 24283470 PMC4282454

[7] Wang Z , Zhao Q, Huang Y, Mo Z, Tian Z, Yang F, Wang Y, Yao L. Wandering spleen with splenic torsion in a toddler: A case report and literature review. Medicine (Baltimore) 2020;99:e22063. 10.1097/MD.0000000000022063. 32925740 PMC7489642

[8] Rafie BA , AbuHamdan OJ, Trengganu NS, et al. Torsion of a wandering spleen as a cause of portal hypertension and mesenteric varices: a rare aetiology. J Surg Case Rep 2018;2018:rjy107.. 29876050 10.1093/jscr/rjy107 PMC5961426

[9] Lane TM , South LM. Management of a wandering spleen. J R Soc Med 1999;92:84–5. 10.1177/014107689909200211. 10450220 PMC1297068

[10] Alghamdi R , Alzahrnai A, Alosaimi A, et al. Infarcted wandering spleen: a case report from Saudi Arabia. J Surg Case Rep 2021;2021:rjab277. 10.1093/jscr/rjab277. 34221345 PMC8245189

